# Demographic Factors Associated with Hantavirus Infection in Bank Voles (*Clethrionomys glareolus*)

**DOI:** 10.3201/eid0809.020037

**Published:** 2002-09

**Authors:** Gert E. Olsson, Neil White, Clas Ahlm, Fredrik Elgh, Ann-Christin Verlemyr, Per Juto, R. Thomas Palo

**Affiliations:** * Swedish University of Agricultural Sciences, Umeå, Sweden; † Umeå University, Umeå, Sweden; ‡ Defense Research Agency, Umeå, Sweden; § Swedish Institute for Infectious Disease Control, Solna, Sweden

**Keywords:** bank vole, *Clethrionomys glareolus*, logistic regression, hantavirus, hemorrhagic fever with renal syndrome, nephropathia epidemica, odds ratio, population density, Puumala virus, rodents

## Abstract

The bank vole (*Clethrionomys glareolus*) is the natural reservoir of *Puumala virus* (PUUV*)*, a species in the genus *Hantavirus*. PUUV is the etiologic agent of nephropathia epidemica, a mild form of hemorrhagic fever with renal syndrome. Factors that influence hantavirus transmission within host populations are not well understood. We evaluated a number of factors influencing on the association of increased PUUV infection in bank voles captured in a region in northern Sweden endemic for the virus. Logistic regression showed four factors that together correctly predicted 80% of the model outcome: age, body mass index, population phase during sampling (increase, peak, or decline/low), and gender. This analysis highlights the importance of population demography in the successful circulation of hantavirus. The chance of infection was greatest during the peak of the population cycle, implying that the likelihood of exposure to hantavirus increases with increasing population density.

The hantaviruses belong to the family *Bunyaviridae* and are the causative agents of hemorrhagic fevers with renal syndrome (HFRS) and hantavirus pulmonary syndrome (HPS) in humans (1,2). Each distinct form of the virus is closely associated with a single, or possibly a few, rodent species (3,4). Transmission of hantaviruses to humans occurs mainly through the inhalation of aerosols containing virus excreted from infected rodents; rodent-to-rodent transmission also may occur through biting and social grooming (5–7). Approximately 150,000 human cases of hantavirus infection are reported per year worldwide (6). Mortality in humans ranges from <0.5% in nephropathia epidemica, a mild form of HFRS (8,9), to 5% to 10% from other HFRS (6), and 45% from the more severe HPS (4).

The only hantavirus isolated in Sweden is the *Puumala virus* (PUUV) from the bank vole (*Clethrionomys glareolus*), which serves as the natural reservoir species. The bank vole is the most common and widespread rodent species in northern Sweden. In northern Fennoscandia, density fluctuations may show up to 500-fold changes from peak to decline/low phase during a 3- to 4-year cycle (10–13). In northern Sweden, the incidence of nephropathia epidemica in humans reaches an average of 40 serologically confirmed cases per 100,000 inhabitants in rodent peak years; yet up to 80% of human cases may be unrecognized (14).

The importance of factors assumed to be associated with the occurrence of hantavirus infections in natural rodent host populations is not well understood (15). Tools need to be developed to model hantavirus transmission in the wild reservoir species to better understand the relationship between the natural circulation of the virus and incidence of the disease in human populations. Among the factors of interest, rodent age and sex are known to distinguish cohorts of high seroprevalence in the wild (16,17). These two factors represent the elapsed time of possible virus exposure and sex-biased behaviors. We have found (18, Olsson et al., mans. in prep.) that higher numbers of PUUV-infected bank voles were associated with sites of known human hantavirus exposure in peak years, suggesting an influence of the local environment on subsequent chance of PUUV exposure. Therefore, sampling sites and phase during population cycle were included in our analysis to evaluate the probability of PUUV infection. We also investigated the influence of the body condition of bank voles on their probability of being PUUV seropositive. The models we considered included a measure of body condition because either 1) malnourished bank voles would be more likely to be PUUV infected because of increased susceptibility (19), or 2) well-nourished bank voles would be more likely to be PUUV seropositive because high-quality habitats support higher or persistent numbers of bank voles (20), facilitating the spread of the virus.

The aim of our study was to relate and rank characteristics of bank voles, i.e., age, body measurements, and sex, influencing the probability of being PUUV seropositive. Complementary independent variables are sampling sites and sampling events within the population cycle. Determining which of these factors is applicable is essential to the modeling of the spread of hantaviruses within a rodent host population.

## Materials and Methods

Sampling of rodents was initiated in the fall of 1995 in the vicinity of three recently affected households in the coastal areas of Västerbotten County in northern Sweden, 63°45´– 63°20´ N, 20°00´– 21°00´ E (18); subsequently, the rodent populations were sampled twice a year, including the fall of 1999. Case sites were denoted “south,” “center,” and “north,” according to location within sampling region, and situated approximately 40 km apart (south-to-north 80 km). At these sites, nephropathia epidemica was serologically confirmed in humans 3–10 weeks before the first sampling event. Collection of animals took place within a 3-week period in May–June and again in September-October each year. Forested area sites randomly situated approximately 10 km from each case site were used as controls and denoted as paired random forest sites. At each of the six sampling sites, 30 snap-traps, baited with dry apple, were placed at 10-m intervals in each of six transects of 300-m length per site. Thus, 180 snap-traps were set during four nights on each site, constituting 720 trap-nights per site of investigation. Trap indices represent success in a sampling effort, as the number of voles captured per 100 trap nights, i.e., a reflection of the relative population density on each sampling occasion. Case sites and random forest sites comprised mainly managed, boreal coniferous forests of Scots Pine (*Pinus sylvestris*) and Norway Spruce (*Picea abies*), with a considerable cover of bilberry (*Vaccinium myrtíllus*) and lingonberry (*V. vitis-idaéa*).

In total, 1,568 bank voles were collected over the 5-year period 1995–1999. The phase of the population development was designated as “increase,” “peak,” or “decline/low,” depending on the relative amplitude of trap indices in consecutive years. Spring and fall samplings were combined because low spring numbers, particularly in 1997, did not allow for separate statistical analysis of seasons.

Bank voles captured in 1995 and 1996 were not available for age determination, but the age of 1,079 bank voles captured in 1997 to 1999 was determined according to criteria of molar root development and growth (21,22); each animal was subsequently assigned to one of four age classes (Table 1). Briefly, lower molar 1 or upper molar 2 was pulled from its socket, placed in a duralumin frame on a microscope slide, and measured to the nearest 0.05 mm under a stereomicroscope. Of the 1,079 specimens, 79 were not measurable on one or several of the other independent variables (Table 1); therefore, the sample size in the following analysis was 1,000 voles. Body condition of bank voles was estimated as the body mass index (BMI = weight/length^2^, [23]), although this BMI can also be considered a measure of animal morphology. Body length measurements were obtained to the nearest millimeter (not including the tail). The total body weight was taken to the nearest 0.01 g. Weights of fetuses from pregnant females were either subtracted, when possible, or excluded from further analyses. We used enzyme-linked immunosorbent assay (ELISA) to detect PUUV immunoglobulin G antibody in bank vole sera. Details of collecting, storing, and processing serologic test results have been described (18,24).

**Table 1:** Binary logistic regression variables included in the model that predicted the probability of a bank vole’s being seropositive to *Puumala virus* (PUUV)

Variable	Variable description	Nominal reference level
Serostatus	Binary response variable, bank voles with PUUV-specific immunoglobulin G antibodies are denoted “1” (“success”) and seronegative bank voles “0” (“failure”)	
Age	Polytomous independent variable, age classes; 1=juvenile/subadult <3 months of age; 2 = adult born in year of sampling 3–6 months of age; 3 = overwintered adult in spring >7 months of age; 4 = overwintered adult in fall >11 months of age	Age class 1
Sex	Dichotomous independent variable for female or male	Females
BMI	Continuous variable body mass index	
Phase	Polytomous independent variable on population phase denoted as increase (1997), peak (1998), or decline/low (1999)	Decline/low
Type	Dichotomous independent variable: case site or random forest site	Random forest site
Pair	Polytomous variable on paired case- and random forest sites located south, center, or north within sampling region	South

Binary logistic regression models with serostatus as the dichotomous response variable were used to identify statistically significant factors and estimate the probabilities of the diagnostic classes, i.e., PUUV seropositive or seronegative (Table 1). We used a logit link function because we wanted to identify the influence of the independent variables separately rather than model the cumulative probability and outcome of each combination of elements. Thus, the analysis prevented confounding effects from any independent variable to the other. We present odds ratios (OR) in favor of infection in relation to reference levels for nominal variables and per unit change for the continuous variable (Table 1). We selected the most parsimonious model by using Akaike’s information criterion (AIC) (25). Statistical analyses were performed by using the R statistical software package release 1.3.0 (http://www.r-project.org), with the methods outlined in Venables and Ripley (26) for generalized linear models of binomial data.

## Results

**Figure 1:**
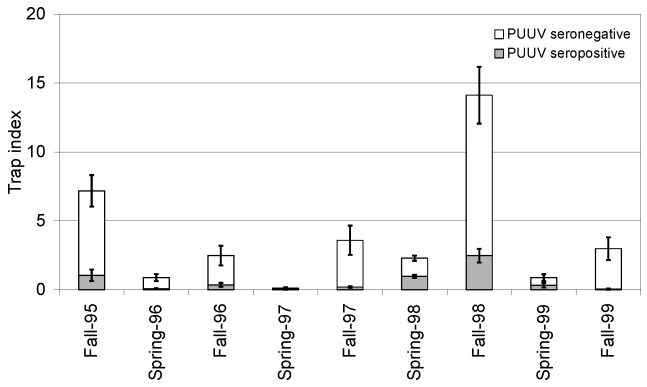
Mean (± SE) trap indices of bank voles in all six sites during a 5-year sampling period in northern Sweden. Trap indices represent success in sampling effort as numbers of voles captured per 100 trap nights. Lower, shaded part of the bars represents trap indices of *Puumula virus* (PUUV)-seropositive bank voles, and the upper part represents PUUV-seronegative voles at each sampling occasion.

The significantly highest trap indices of bank voles were recorded in falls of 1995 and 1998, and lower indices of bank voles were recorded during the other sampling occasions (Figure 1; Olsson et al., unpub. data). The overall PUUV seroprevalence was 15.4% in the bank voles sampled during the study period, 1995–1999.

Results from two models of logistic regression are presented (Table 2). Model I included six independent variables (age, gender, BMI, and the population phase, type, and pair), and model II included all but the nonsignificant variables “type” and “pair.” The response variable in each of the models was the individual bank vole’s PUUV serostatus (seropositive 1; seronegative 0). Interactions between independent variables were examined but found to be nonsignificant. The two models were accepted as appropriate on the basis of the goodness-of-fit test. In model II, two of the independent variables used in model I were excluded because they did not significantly contribute to the outcome of the analysis; the type of sample site denoted as “case,” “random forest,” and “pair” (north, south, and center) indicated geographic location within the sampling region. The AIC method, which suggests the direction towards selecting the best model, was used to compare and select the most parsimonious set of independent variables. AIC favored model II, which predicted 80.4% (AIC 674.999) of the responses correctly, compared with 80.7% for model I (AIC 679.897).

**Table 2:** Outcome of the binary logistic regression models I and II predictions of risk of a bank vole’s being seropositive for *Puumula virus* under specified conditions in relation to reference levels ^a–f^

	Model I					Model II				
Predictor	Coeff.	Z^g^	p^h^	OR	95% CI	Coeff.	*Z*	p	OR	95% CI
Age^a^										
2	1.71	4.69	<0.001	5.55	2.71 to 11.36	1.74	4.76	<0.001	5.67	2.78 to 11.6
3	1.43	5.03	<0.001	4.18	2.39 to 7.3	1.39	5.03	<0.001	4.01	2.33 to 6.89
4	3.41	6.73	<0.001	30.27	11.22 to 81.7	3.36	6.69	<0.001	28.7	10.7 to 76.6
Gender^b^										
Male	0.61	2.72	0.007	1.84	1.19 to 2.87	0.62	2.78	0.005	1.87	1.2 to 2.9
BMI^c^	1.39	3.43	0.001	4.03	1.82 to 8.94	1.41	3.48	<0.001	4.1	1.85 to 9.07
Phase^d^										
Peak	0.88	2.57	0.01	2.4	1.23 to 4.7	0.87	2.58	0.01	2.4	1.23 to 4.65
Increase	–1.03	–2.07	0.04	0.36	0.13 to 0.95	–1.03	–2.1	0.04	0.36	0.14 to 0.93
Type^e^										
Case site	0.10	0.48	0.63	1.11	0.73 to 1.69					
Pair^f^										
Center	0.17	0.67	0.50	1.19	0.72 to 1.95					
North	0.21	0.88	0.38	1.24	0.77 to 2.0					
^a^The age class with the largest number of individuals was set as reference with which all others were compared, i.e., age class 1 (juveniles and subadults). ^b^Female bank voles were set as reference. ^c^Body mass index (BMI) is a continuous variable; OR refers to unit increase. ^d^The decline/low population phase was used as reference. ^e^The sampling area type “random forest site” was set as reference. ^f^South sampling pair was reference to other pairs on regional effect. ^g^The Z-score shows the number of standard deviations that the tested predictor class’s coefficient falls above or below the predictor’s reference level. ^h^The p value is the probability that the observed coefficient of the actual predictor class’s should be by random chance variation. Coeff., coefficient; OR, odds ratio; CI, confidence interval.										

**Figure 2:**
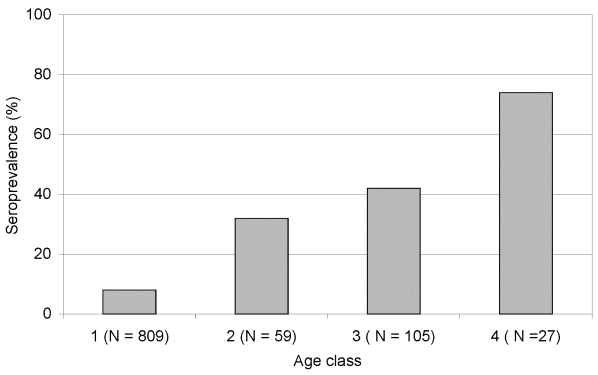
Seroprevalence of *Puumula virus* (PUUV)-specific immunoglobulin G antibodies within different age classes of bank voles. 1 = juvenile/subadult <3 months of age; 2 = adult born in year of sampling 3–6 months of age; 3 = overwintered adult in spring >7 months of age; 4 = overwintered adult in fall >11 months of age.

The importance of age as a transmission factor is shown by the high OR for the oldest age class (Table 2; Model II). The interpretation of this OR would be that the odds of being seropositive in age class 4 was 28.7 times greater than in age class 1. This value was the highest in the model, followed by that of age class 2 (OR 5.67). For age class 3, the OR was similar (4.01). The age-related increase in chance of being PUUV seropositive was also observed for seroprevalence, i.e., proportion seropositive, within each age class (Figure 2).

**Figure 3:**
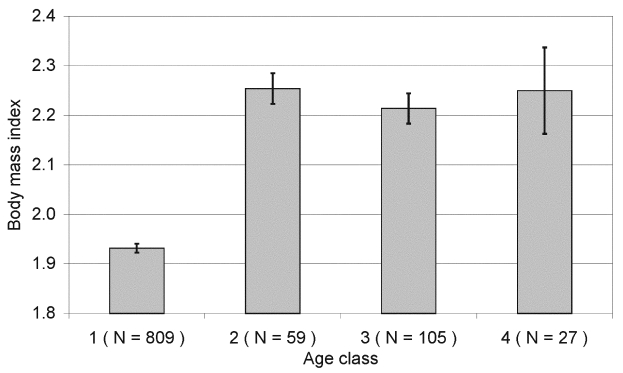
Mean (± SE) body mass index (BMI) within age classes of bank voles, where BMI separates age class 1 (juvenile/subadult) from all others; see Figure 1 for details on age classes.

BMI was the second most important factor based on the rank of OR. Each unit increase in BMI led to a 4.1 times higher probability that a bank vole was seropositive. Age may indirectly play a role here because BMI within age class 1 differed considerably from that in the other age classes; however, the difference between age classes ranged within 0.4 units (Figure 3).

The phase of the population development (increase, peak, or decline/low) during which the bank vole was sampled was also a significant factor (Table 2). For bank voles captured during the peak in the population cycle, the OR in favor of being seropositive were 2.4 higher than in decline/low phase (p=0.01). The OR during the increase phase was 0.36 (p=0.04), indicating that chance of being seropositive was actually higher during the decline/low phase than during the increase. The fourth independent variable in rank was the animal’s gender (Table 2). Overall, the odds in favor of infection for males were 1.87 times higher than for females.

## Discussion

The population abundances of bank voles varied, with 3- to 4-year fluctuations, as previously described for microtine rodents in northern Scandinavia, as did the intra-annual abundances, with higher population densities in fall than in spring (11,12). The classification of sampling occasions as increase, peak, and decline/low therefore appears to be appropriate (Table 1).

The factor with the highest influence on the probability of being PUUV seropositive was the vole age at capture. Age is an important epidemiologic parameter because chance of exposure to horizontally transmitted pathogens generally increases with age (27,28). The overwintering bank voles captured in the fall, i.e., age class 4, constituted at the most 3.6% of the total sample in the increase and peak years; however, three of four of these specimens were PUUV seropositive, constituting a small but important virus reservoir (Figure 2). No captures of that cohort were made in the decline/low years, when, on the whole, fewer seropositive specimens were captured. Our findings appear to agree with the hypothetical model of Mills et al. (16), in which the effect of consecutive years of favorable conditions on population growth and consequent hantavirus transmission is considered one of the most important factors. In the model, the proportion of overwintering adults remained the same under the extended favorable conditions, but absolute numbers increased, thus facilitating the limited winter transmission to susceptible voles within the population. The bank vole population fluctuations in our study appear to be cyclic, and the increase and peak phases resemble the favorable circumstances for the hantavirus host population, as discussed by Mills et al. (16). The mass action principle of disease transmission assumes that transmission is a function of the densities of infectious and susceptible animals (29). However, because of the rapidly diminishing densities of bank vole populations after the peak in the trapping index each fall, we suggest that opportunities for hantavirus transmission decrease drastically and therefore very likely deter any time-lagged density dependence on hantavirus prevalence. In addition, as the logistic regression model shows, the studied populations were not homogeneously mixed across the demographic delimitations (e.g., age groups and gender), as the individual bank voles showed no random chance of being PUUV seropositive. Therefore, the mass action principle is not applicable to the studied system.

Communal nesting is beneficial to overwintering bank voles (30,31) and may be a facilitating factor on the successful persistence of PUUV in local populations during the nonbreeding season. However, the frequent contacts between sexually mature voles during the breeding season are likely to be a situation more critical to hantavirus transmission. The difference in OR between age classes 2 and 3 was nonsignificant, as shown by the almost completely overlapping 95% confidence intervals, suggesting that transmission between overwintering specimens (living through fall to spring) was limited in the studied populations. However, the difference between age classes 3 and 4 was highly significant, as shown by the separation in confidence intervals, indicating that adult bank voles that survived the winter and thus engaged in breeding the following summer were subject to considerable hantavirus exposure during that breeding season. These patterns likely reflect the frequency and degree of social interactions among bank voles during the winter versus the summer breeding season. The behavioral mechanism facilitating hantavirus transmission associated with sexual maturation is probably also applicable to age class 2, i.e., the specimens that likely became sexually mature during the current breeding season. This association is supported by the similar observations of Glass et al. (32) on the seroprevalence in sexually maturing Norway rats (*Rattus norvegicus*). Bernshtein et al. (17) also observed an increased rate of hantavirus transmission in bank voles during the period of high reproductive activity.

BMI, the second most influential factor, was originally developed to measure obesity in humans (23). We used BMI to identify and separate malnourished bank voles from well-nourished ones and their chances of PUUV infection. BMI reflected the distribution within age classes in that age class 1, i.e., juveniles and subadults <3 months of age, had a lower BMI than all other age classes, although the method of logistic regression rules out age per se as the confounding effect (Figure 3). One likely explanation for the differences in BMI is the changes in morphology associated with sexual maturation. As the range in BMI appeared not to overlap between the juveniles and subadults of the year (<3 months of age) and the adults born in the year of sampling (3–6 months of age), BMI may serve as a tool to distinguish the two cohorts in similar studies, when estimating the age by using the techniques discussed here may be impractical.

The effect of the peak year suggests a direct vole density–dependent chance of PUUV infection. As the chance of being PUUV seropositive in the peak of the population cycle was 2.4 times higher than in the decline/low, we conclude that this effect is caused by the density of the bank vole population. Other researchers have proposed that delayed density dependence occurs (16,33). Since the chance of infection was lower in the increase phase than the decline/low phase, we suggest that the effect was not so much a result of delayed density dependence but of having a large reservoir of infection in the population.

Results from other hantavirus-rodent systems also show a sex-related bias in the odds of infection similar to our observations. This effect is likely caused by differences in behavior between males and females (16,17). Aggressive behavior, such as biting, between males has been suggested as a means of hantavirus transmission in other studies (32). However, males of *Clethrionomys* spp. do not defend territories but usually have overlapping home ranges in their competition for mating. Females do, however, compete for food and defend territories (20,34,35). Therefore, the gender effect in serostatus more likely emerges because males are more labile and subsequently have more frequent encounters with conspecifics, increasing their chance of contracting the virus.

In conclusion, long-lived bank voles appear critical to the success of hantavirus circulation and persistence within host populations. Localized absence of PUUV coincided with the absence of overwintering specimens at several sites during population decline/low. The chance of being PUUV seropositive is related to phase of population cycle and is therefore density dependent. Quantitative measures like these revealed by logistic regression are useful in developing demographic host models on the subsequent risks of exposure to humans in areas of critical rodent host dynamics.
